# Supplementary material for: The histologic phenotype of lung cancers is associated with transcriptomic features rather than genomic characteristics
Source: Nat Commun. 2021 Dec 6;12:7081. doi: 10.1038/s41467-021-27341-1 (PMC8648877; doi:10.1038/s41467-021-27341-1)
Supplement: Supplementary file 1 — Supplementary Information [file 41467_2021_27341_MOESM1_ESM.pdf]

Supplemental Information

The histologic phenotype of lung cancers is associated with transcriptomic features rather than genomic characteristics

Tang, Hussein et al.

Supplementary Fig. 1

a

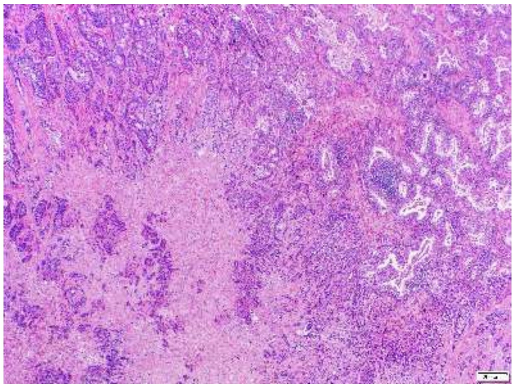

b

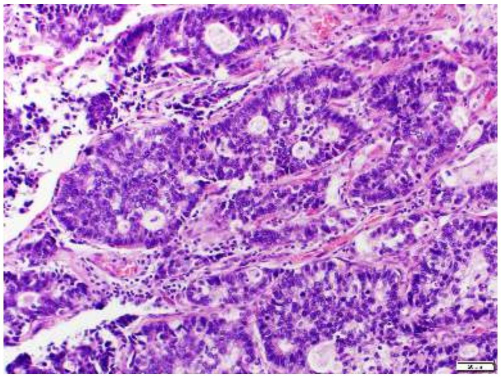

c

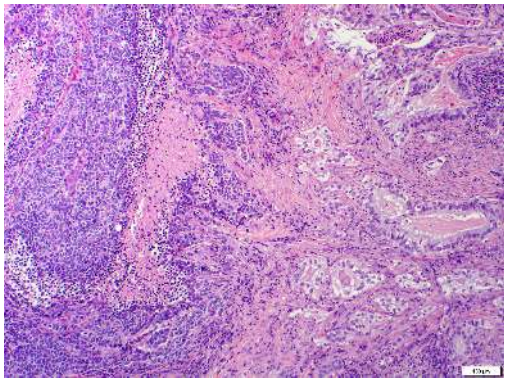

d

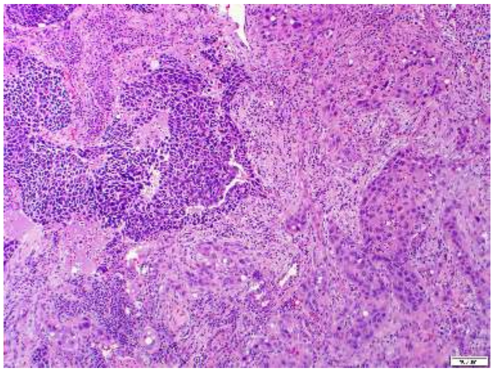

Supplementary Fig 1. Representative H&E stained sections of tumors in patients 30, 35, and 37. Each sample was stained for 3 sections. (a). Low magnification image of mixed adenocarcinoma (right) and large cell neuroendocrine carcinoma (left) for Pa30. (b) High magnification image of the large cell neuroendocrine carcinoma (LCNEC) component for Pa30. (c) The image shows a mixed tumor encompassing moderately differentiated adenocarcinoma (right) and small cell carcinoma (left) for Pa35. (d) The image captures a mixed tumor composed of moderately differentiated squamous cell carcinoma (right) and small cell carcinoma (left) for Pa37.

Supplementary Fig. 2

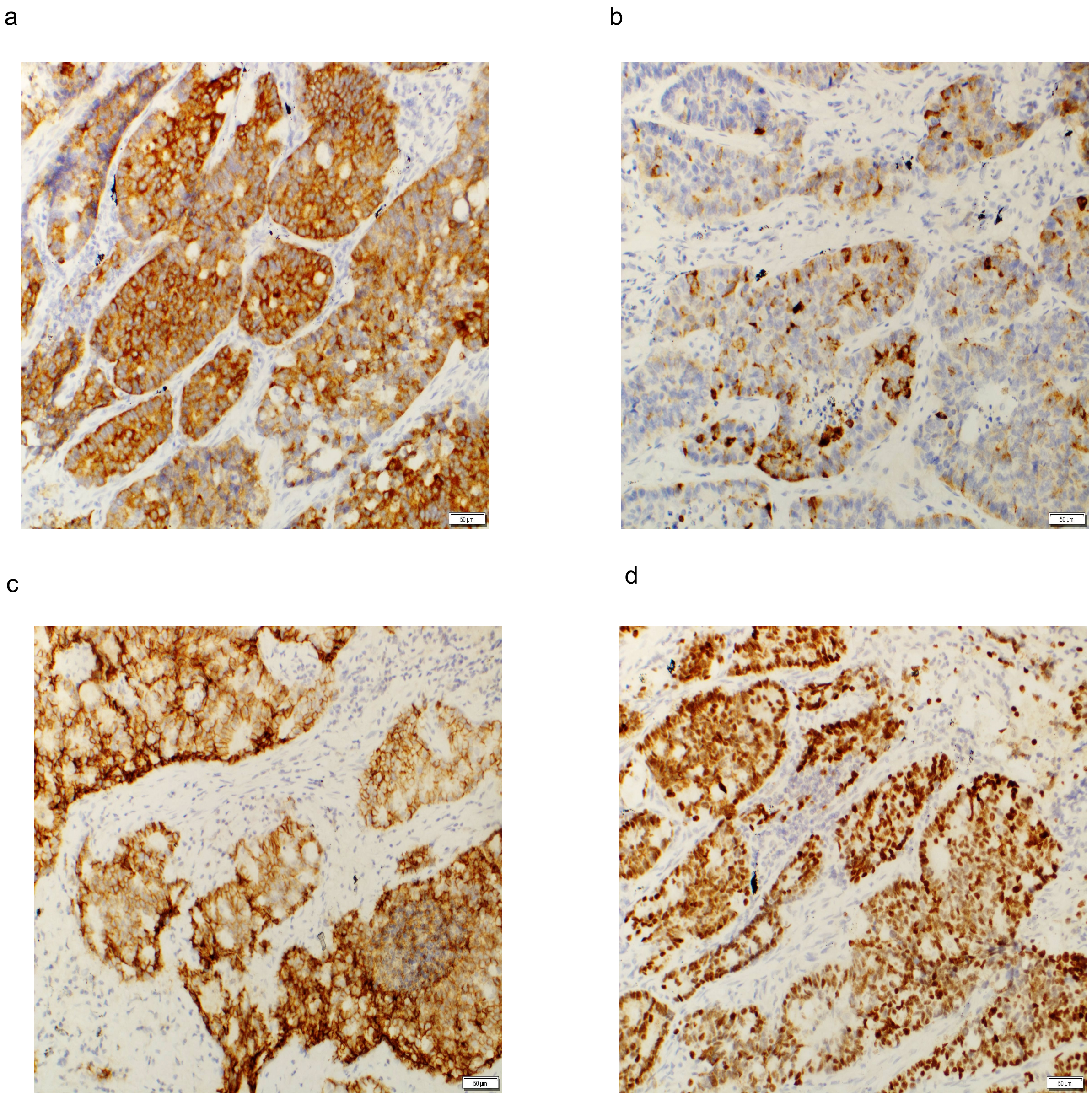

Supplementary Fig 2. Immunohistochemical stains corresponding to the LCNEC component of Pa30 (a) Synaptophsin (b) Chromogranin (c) CD56 (d) TTF-1. Each sample was stains for 3 sections.

Supplementary Fig. 3

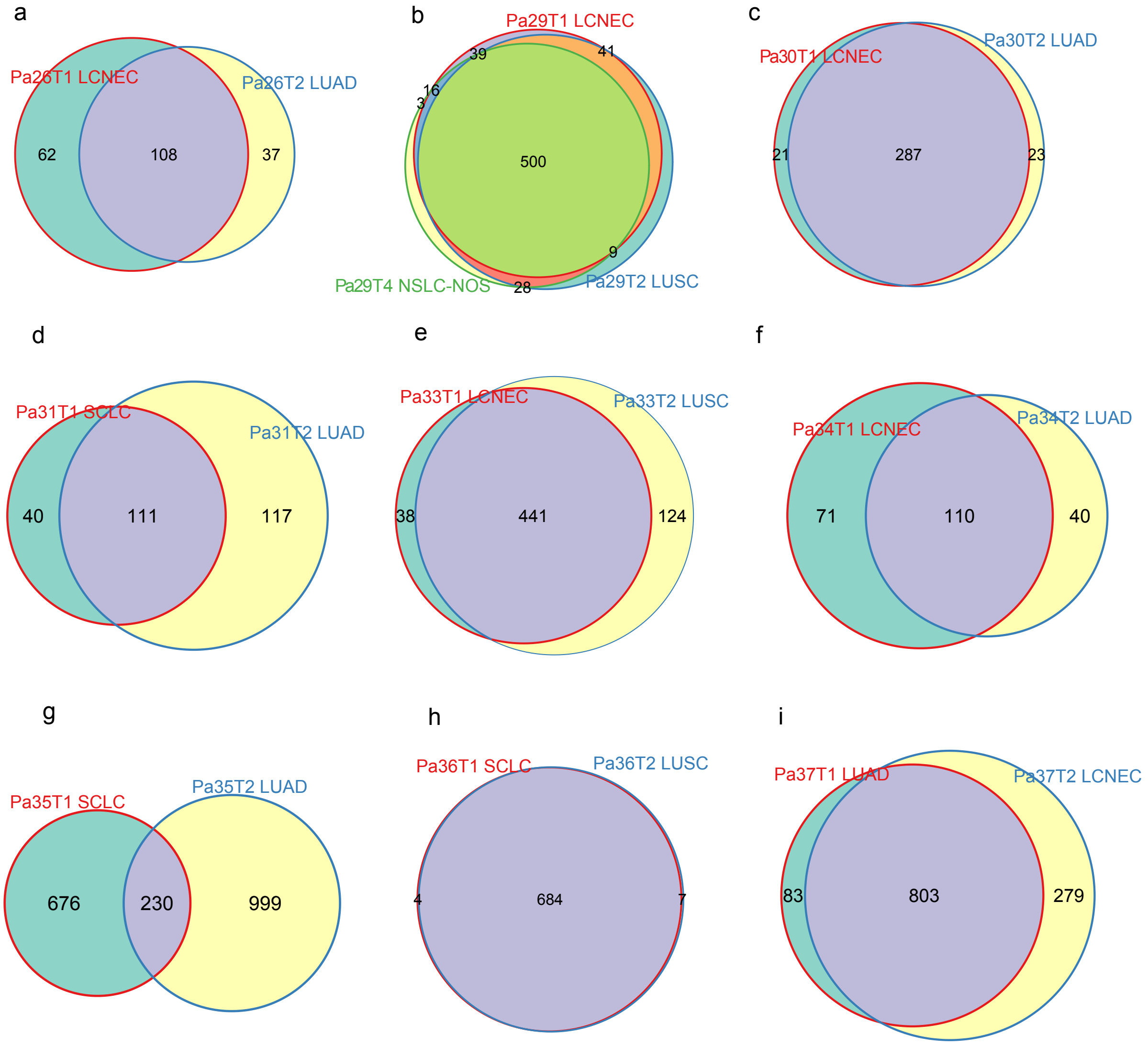

Supplementary Fig 3. Overlapping mutation number between different histologic components within the same patient. (a-i) Venn-diagram showing the number of overlapping mutations in each patient across different histological components. Source data are provided as a Source Data file.

Supplementary Fig. 4

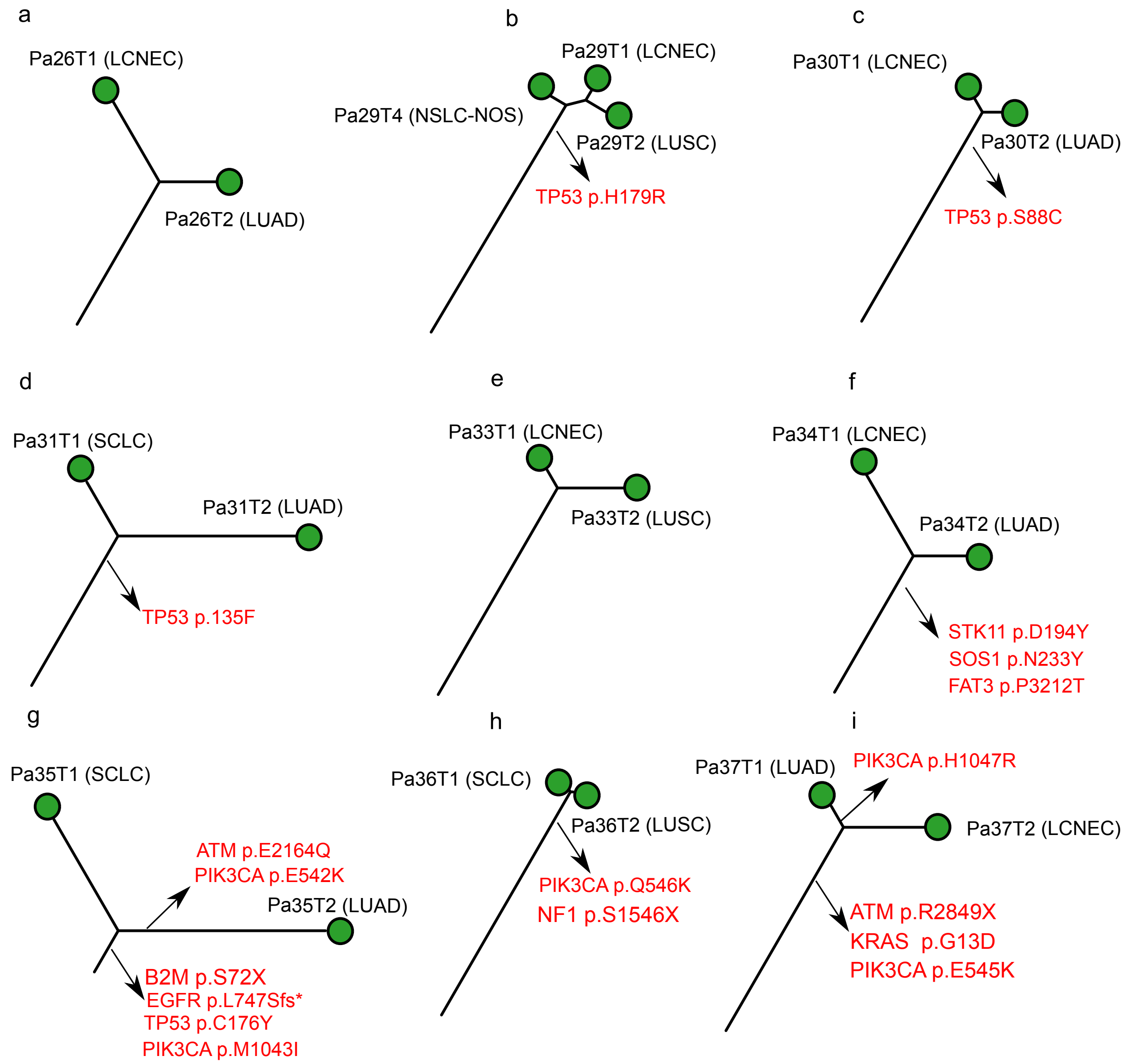

Supplementary Fig 4. Phylogenetics trees of different histologic components within the same patient. (a-i) Lengths of trunks and branches are proportional to the number of somatic mutations. Canonical cancer gene mutations are mapped to trunk versus branches respectively. Source data are provided as a Source Data file.
