# Supplementary material for: The histologic phenotype of lung cancers is associated with transcriptomic features rather than genomic characteristics
Source: Nat Commun. 2021 Dec 6;12:7081. doi: 10.1038/s41467-021-27341-1 (PMC8648877; doi:10.1038/s41467-021-27341-1)
Supplement: Supplementary file 3 — Description of Additional Supplementary Files [file 41467_2021_27341_MOESM3_ESM.pdf]

File name: Supplementary Data 1

Description: Patient information

File name: Supplementary Data 2

Description: All somatic mutation calls for all samples

File name: Supplementary Data 3

Description: Cancer driver mutation calls for all samples

File name: Supplementary Data 4

Description: RB1 LOH status for all samples

File name: Supplementary Data 5

Description: Pathway enrichment analysis for public and in-house gene expression data
